# Fundamental Limitation: How Through-Thickness Heterogeneity in Laser-Reduced Graphene Oxide Compromises Sensor Stability

**DOI:** 10.1021/acsanm.6c00918

**Published:** 2026-05-07

**Authors:** Ryan Russell, Alex Medeiros, Aiden Rowley, Sandra Schujman, Ivan V. Vlassiouk, Timothy J. Barnum, Yijing Stehle

**Affiliations:** † Department of Mechanical Engineering, 7254 Union College, Schenectady, New York 12308, United States; ‡ NYCREATES, Albany, New York 12203, United States; § Center for Nanophase Materials Sciences, Oak Ridge National Laboratory, Oak Ridge, Tennessee 37831, United States; ∥ Department of Chemistry, 7254 Union College, Schenectady, New York 12308, United States

**Keywords:** graphene oxide, laser reduction, reduced graphene oxide (rGO), humidity sensing, material heterogeneity, sensor stability, top-down fabrication

## Abstract

Laser-induced reduction of graphene oxide (GO) is a promising technique for the rapid, single-step fabrication of porous carbon electrodes for electronic devices. However, the translation of this method into reliable, high-performance applications is often constrained by a lack of understanding of the material’s inherent structural and chemical heterogeneity. This study presents a parametric investigation into the pulsed CO_2_ laser reduction of free-standing GO membranes to deconvolve the relationship between processing parameters, material properties, and device stability. Through detailed morphological (SEM), chemical (XPS, FTIR), and structural (XRD, Raman) characterization, we identify the optimal laser power (2.4 W) to produce a highly porous, “peony-like” rGO architecture with a high surface area of 234 m^2^/g. Crucially, we provide definitive evidence of a “top-down” reduction mechanism, where high-quality lattice restoration via thermal disproportionation is restricted to the surface layers. This inherent through-thickness heterogeneity creates a “buried” hydrophilic reservoir that acts as a functional bottleneck, leading to the observed baseline drift and moisture-driven instability in sensing applications. The residual GO layer strongly absorbs water molecules, leading to an extremely slow and incomplete recovery, rendering the material unsuitable for practical sensing applications. These findings highlight a fundamental limitation of direct laser writing for creating uniform functional materials and underscore the critical role that through-thickness heterogeneity plays in device performance and stability.

## Graphical abstract

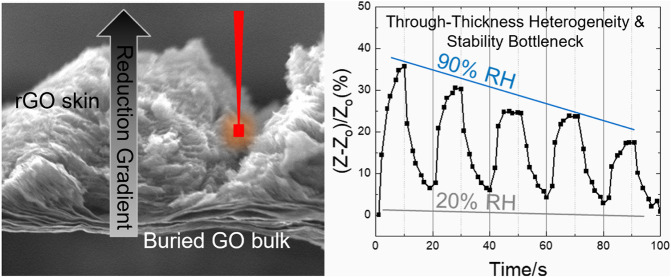

## 1 Introduction

Graphene, a single layer of carbon atoms in hexagonal lattice, has garnered scientific interest for its exceptional physical properties, most notably its extremely high charge carrier mobility of 200,000 cm^2^/V·s.^1,2^ This makes it a prime candidate for next-generation electronic devices. However, the practical application of pristine graphene is severely inhibited by its poor processability and insolubility in common solvents, making large-scale manufacturing and integration challenging.^3–5^ To overcome these processing limitations, graphene oxide (GO) was developed as a tractable precursor. GO is a graphene sheet decorated with oxygenated functional groups (e.g., hydroxyl, epoxy, carboxyl), which render it hydrophilic and dispersible in water. This ease of processing comes at a cost: the disruption of the pristine lattice makes GO an electrical insulator, limiting its direct use in electronics.^6,7^ Consequently, a reduction step is essential to remove these oxygen groups, partially restore the conductive sp^2^ carbon network, and produce reduced graphene oxide (rGO). While rGO’s enhanced conductivity makes it suitable for applications like supercapacitors, sensors, and batteries, the reduction is typically incomplete, leaving residual oxygen and structural defects that prevent it from matching the quality of pristine graphene.^8,9^

A variety of methods exist to reduce GO, including chemical, thermal, and electrochemical approaches.^10–15^ Each method offers distinct advantages and disadvantages in terms of scalability, cost, environmental impact, and the resulting rGO quality. While chemical reduction routes are common, they often introduce impurities and permanent lattice defects. Thermal processing, specifically through laser-induced pathways, offers a cleaner alternative that can potentially preserve the hexagonal long-range order of the carbon framework. Among these, laser-induced reduction has emerged as a particularly promising technique. Laser processing offers several distinct advantages: it is a rapid, single-step, and chemical-free method that allows for the direct, mask-less patterning of GO films with high precision. This process can be performed in ambient conditions and can simultaneously create a highly porous morphology in the resulting rGO, which is beneficial for enhancing ion accessibility in electrochemical devices. This type of highly porous rGO shows promising performance when applied as electrodes of supercapacitors,^15–17^ and sensing devices.^18–21^ LrGO has been used in the fabrication of flexible sensors, such as flexible strain sensors,^18^ thermal sensor,^19^ gas sensors,^20^ and pressure sensors.^21^

Despite these advantages, the long-term functional stability of laser-reduced GO (LrGO) devices remains a critical challenge. Current literature often overlooks the vertical chemical gradients produced by top-down irradiation. This study specifically investigates how this through-thickness heterogeneity impacts device performance, providing a mechanistic link between laser-matter interactions and sensor reliability.^22–25^ Specifically, two critical, interconnected problems persist. (1) Through-thickness heterogeneity: the reduction process is governed by the absorption of laser energy, which attenuates exponentially with penetration depth. This leads to a pronounced reduction gradient through the thickness of the GO film. The top surface, directly exposed to the laser, may achieve a high degree of reduction, while the underlying layers remain partially or entirely unreduced. This results not in a uniform rGO film, but in a heterogeneous composite structure. The presence of this hydrophilic, electrically insulating residual GO at the substrate interface is particularly detrimental, as it can absorb ambient moisture, leading to poor environmental stability and unpredictable drift in the electrical performance of devices. (2) The deoxygenation-defect trade-off: achieving a low oxygen-to-carbon (O/C) ratio, which is essential for high electrical conductivity, requires aggressive laser parameters (high power or low scan speed). However, the intense localized heat generated during this process can cause irreparable damage to the graphene lattice. This thermal shock can introduce new structural defects—such as vacancies, pores, and bond distortions—or even cause ablation of the material. These process-induced defects act as scattering sites for charge carriers, ultimately placing an upper ceiling on the achievable conductivity. Therefore, a delicate balance must be struck between maximizing oxygen removal and minimizing damage to the restored sp^2^ carbon framework, a balance that is not yet well understood or controlled.

This study aims to directly address these challenges through a parametric investigation of the pulsed CO_2_ laser (10.6 μm) reduction of GO membranes. We seek to deconvolve the complex interplay between processing parameters (power, speed, frequency) and the resulting material properties by optimizing for a single, critical performance metric: electrical conductivity. To isolate the properties of the optimally reduced material from the influence of the heterogeneous bulk film, we employ a novel comparative approach, analyzing both the “direct” as-fabricated LrGO film and a “recollected” LrGO powder derived from its well-reduced top surface. The structural and chemical evolution is comprehensively characterized to elucidate the mechanisms and limitations of the reduction process. Finally, by subjecting the optimized electrodes to varying humidity conditions, we directly quantify the impact of through-thickness heterogeneity on device stability. The findings provide a deeper understanding of the intrinsic limitations of photothermal reduction and offer a pathway toward fabricating more reliable and higher-performance LrGO-based electronic and electrochemical devices.

## 2 Experimental Section

### 2.1 Materials and Fabrication

Graphene oxide (GO) aqueous dispersion (4 mg/mL, monolayer content >95%) was purchased from Graphenea (Cambridge, UK). The GO was synthesized via a modified Hummers’ method, ensuring a high density of oxygen-containing functional groups (OFGs) for subsequent reduction processing. Free-standing GO membranes were fabricated by casting 70 g of the as-received dispersion into a 120 mm diameter Teflon dish, followed by evaporation-induced self-assembly under ambient conditions (25 °C).^26^ The resulting membranes exhibited a spatial thickness variation between 10 and 16 μm across the 113 cm^2^ area, as characterized by a Positest Coating Thickness Gage and verified through cross-sectional Scanning Electron Microscopy (SEM). This thickness range was maintained for all subsequent laser-patterning experiments to ensure consistency in photonic energy absorption through the membrane bulk. For laser irradiation, the GO membrane was mounted on a flat microscope glass slide to ensure a uniform focal plane. Laser treatment was performed using a modulated CO_2_ laser system (Epilog Fusion Pro, wavelength: 10.6 μm) operating in a raster-scan mode under ambient conditions. To maintain a constant energy delivery profile across all samples, the scan speed was fixed at 100% (equivalent to 3048 mm/s), and the modulation frequency was set to 20%. The irradiation profile was defined by a horizontal raster pattern with a resolution of ∼200 DPI, resulting in a controlled interdot spacing of 130 μm. The laser power was the primary variable, ranging from 1.2 to 3.6 W. All patterns were generated using a single pass with the beam focused on the top surface of the membrane (spot size ∼100 μm). When varying the laser power, all other kinematic and modulation parameters (speed, frequency, and DPI) were held constant to ensure the observed effects were solely power-dependent.

### 2.2 Material Characterization

Surface morphology was characterized using a Zeiss EVO SEM at an accelerating voltage of 10 kV utilizing a secondary electron (SE) detector. Elemental analysis was performed using an attached Energy Dispersive Spectroscopy (EDS) detector. X-ray Diffraction (XRD) patterns were recorded using a Rigaku SmartLab X-ray diffractometer with Cu Kα radiation (λ = 0.15418 nm). Scans were performed in reflection mode from 2θ = 5–40° at a rate of 2°/min. The interlayer *d*-spacing was calculated using Bragg’s law. Raman spectra were collected using a Bruker RAMAN Spectrometer equipped with a 785 nm laser excitation source. To minimize the risk of sample degradation or unintended laser-induced reduction during measurement, the laser power at the sample surface was kept below 1 mW. Data were averaged over 10 accumulations with an integration time of 10 s per scan to ensure a high signal-to-noise ratio. The wettability of the GO and rGO surfaces was assessed by measuring the static water contact angle at 21 °C with a Biolin Scientific Optical Tensiometer using the Young–Laplace fit. For each sample, five 10 μL deionized water droplets were analyzed, with data recorded at 0.68 FPS for 10 s to ensure equilibrium. Thermogravimetric analysis was performed using a TGA 8000 FTIR/NIR Spectrometer (PerkinElmer). Approximately 1 mg of square membrane sample was analyzed under a nitrogen (N_2_) atmosphere to prevent oxidation. The temperature was increased from 30 to 600 °C at a constant rate of 10 °C min^–1^. The nitrogen flow rate was maintained at 20 mL min^–1^ throughout the experiment to ensure a stable and inert environment. Infrared (FTIR) spectra of the membranes were taken using a Nicolet iS5 FTIR Spectrometer. X-ray photoelectron spectroscopy (XPS) analysis was performed using a Physical Electronics Quantera Hybrid instrument. A monochromatic Al K-alpha X-ray source (1486.6 eV photon energy) was utilized with an operating power of 50 W, 15 kV, and a 200 μm beam size. An electron neutralizer and an Ar^+^ ion gun was simultaneously employed for charge compensation during the analysis of samples with insufficient conductivity. Survey spectra were acquired with a pass energy of 280 eV, while high-resolution spectra were collected with a pass energy of 55 eV. Angle-resolved measurements were carried out at takeoff angles of 20°, 45° and 75° using a narrow aperture slit for the analyzer.

### 2.3 Electrochemical and Sensor Testing

The conductivity of LrGO patterns was measured using a CHI 680 electrochemical workstation, with Electrochemical Impedance Spectroscopy (EIS) performed from 0.01 Hz to 100 kHz in an ambient lab environment (∼25 °C). A fixed bias of 0.005 V at a frequency of 1000 Hz was applied, with a sampling interval of 1 s. The electrode configuration utilized the unreduced GO bulk as an integrated dielectric separator between the laser-patterned LrGO electrode lines. The response to relative humidity (RH) was evaluated in two modes. For static measurements, controlled humidity environments were created in sealed enclosures using saturated salt solutions: LiCl (11% RH), MgCl_2_ (33% RH), Mg­(NO_3_)_2_ (54% RH), NH_4_NO_3_ (67% RH), and (NH_4_)_2_SO_4_ (81% RH) at 25 °C.^27,28^ An Arduino DHT22 sensor monitored the RH inside the enclosure. For dynamic sensing tests, electrodes were cyclically exposed to a high humidity environment (90% RH from water vapor) for 10 s, followed by a recovery period in ambient lab conditions (∼20% RH) for 10 s.

## 3 Results and Discussion

### 3.1 Morphology of Top-Down Reduction of GO under Laser Irradiation

The morphological transformation of the graphene oxide (GO) film as a function of increasing laser power was investigated using scanning electron microscopy (SEM), as shown in Figure 1. The images reveal a distinct evolution from initial surface modification at low power to the formation of a porous three-dimensional structure at optimal power, and finally to material ablation at high power.

**Figure 1:**
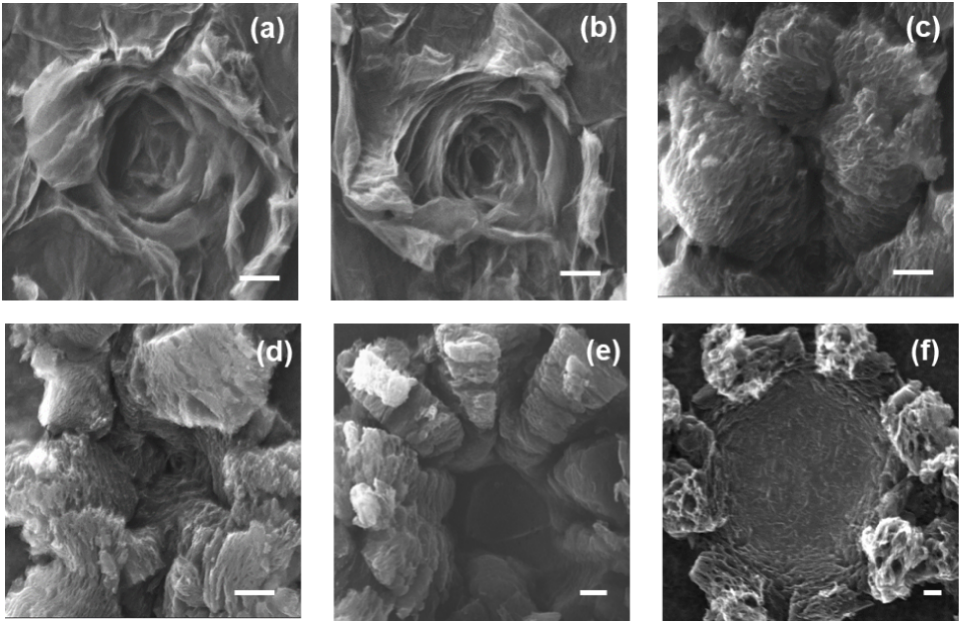
SEM image of laser reduced dots with laser power of (a) 1.2 W, (b) 1.8 W, (c) 2.4 W, (d) 3 W, (e) 3.6 W, and (f) 4.2 W at the frequency of 20% (scale bar 20 μm).

At a low laser power of 1.2 W (Figure 1a), the photothermal reduction process is initiated. The rapid removal of OFGs as gaseous species (e.g., CO, CO_2_, H_2_O) creates internal pressure between the GO sheets. This pressure causes the film to delaminate and the individual sheets to exfoliate. Analogous to the gas release that causes a pastry to rise during baking, this rapid expansion forms a “popped up” structure. The effect is most pronounced on the top layers, which are directly exposed to the laser beam, resulting in larger openings and cracks. Deeper layers are less affected, exhibiting minimal structural change. Increasing the power to 1.8 W (Figure 1b) intensifies this process, leading to the delamination of a greater number of layers and a more pronounced vertical expansion of the reduced region.

A laser power of 2.4 W (Figure 1c) was found to be optimal for creating a well-defined, three-dimensional porous architecture. The structure is characterized by a “peony-like"” morphology, with thin, interconnected sheets of rGO forming a hierarchical network of micropores. This highly porous structure, consistent with previous reports,^7,15^ is ideal for electrode applications as it provides a large surface area and facilitates efficient ion transport. This structural transformation is corroborated by elemental analysis, which shows a significant decrease in the oxygen-to-carbon (O/C) ratio from ∼0.4 in pristine GO to a minimum of ∼0.2 in the rGO region (Figure S1a). This confirms that 2.4 W is the most effective power setting for the deoxygenation of GO without causing significant material damage.

Further increasing the laser power beyond this point leads to thermal damage and material loss. At 3.0 W (Figure 1d), the delicate porous structure begins to fracture, creating larger peony-like splits. Paradoxically, the O/C ratio begins to slightly increase at this power (Figure S1b). This is attributed to the onset of preferential thermal ablation, where the high-energy density promotes the oxidative removal of sp^2^-bonded carbon atoms over the residual oxygen-containing functional groups (OFGs), or the localized reaction of heated carbon with atmospheric oxygen. This shift does not represent a chemical reoxidation but rather a mass-loss regime where the carbon framework is volatilized faster than the remaining oxygen species. At 3.6 W (Figure 1e), the laser power is sufficient to ablate material through the entire thickness of the GO membrane, creating a hole. This is confirmed by elemental analysis (Figure S1c), which indicates the removal of carbon atoms. At 4.2 W (Figure 1f), the area of ablation expands, leaving behind a smaller amount of damaged LrGO around the periphery of the hole.

Based on these results, a laser power of 2.4 W was identified as the best power level under the fixed kinematic and modulation settings studied here. This power level achieves the most effective reduction (lowest O/C ratio) while simultaneously generating a desirable, highly porous 3D morphology. It strikes a balance between sufficient energy for reduction and exfoliation without inducing destructive thermal ablation. Therefore, all electrodes for subsequent testing were fabricated using these laser parameters.

The peony-like morphology (Figure 1c) raises a fundamental question about its formation: does the entire stack of GO layers expand collectively and simultaneously, like a blooming peony, or does it form through a rapid, sequential wrapping of individual sheets as the laser energy penetrates downward? To distinguish between these two potential mechanisms, a simple experiment was conducted. A single LrGO structure was isolated by sonicating the sample in water for 1 min. Pristine GO is hydrophilic and readily disperses into individual sheets under sonication. In contrast, the laser-reduced GO has fewer hydrophilic oxygen functional groups, making it more hydrophobic and stable against complete dispersion. This procedure effectively detaches the LrGO “peony” from the underlying, unreacted GO film, allowing for detailed inspection of its structure.

Figure 2 presents a schematic of the laser interaction (left panel) and an SEM image of a resulting isolated LrGO particle after sonication (right panel). The SEM image provides clear evidence for the formation mechanism. Rather than a uniformly expanded structure with aligned layers, the particle consists of numerous, thin LrGO sheets that are crumpled and wrapped in various orientations. This misorientation between adjacent layers strongly supports the mechanism of rapid, sequential delamination. Each sheet, upon receiving sufficient thermal energy from the laser, wraps and crumples independently of its neighbors. This process happens so quickly that the sheets form a tangled, yet stable, three-dimensional structure rather than a neatly expanded stack.

**Figure 2:**
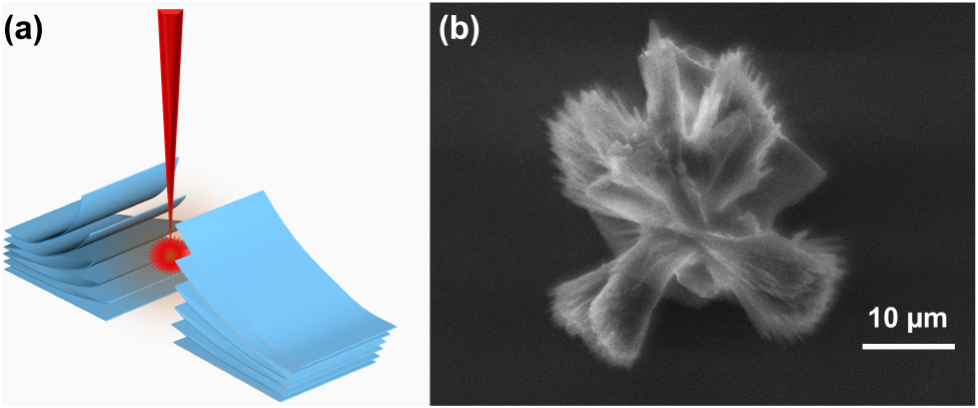
(a) Schematic of the laser-induced exfoliation and (b) SEM image of the isolated laser-reduced GO structure.

By optimizing the laser’s repetition and scan rate, the individual laser-reduced dots can be stitched together to form continuous, conductive lines. Using a power of 2.4 W well-defined LrGO lines were fabricated, as shown in the SEM images in Figure 3. The top-down view (Figure 3a) reveals that the individual peony-like structures are formed sequentially and are well-aligned along the laser’s scanning path. More importantly, these images provide direct visual confirmation of the “top–down” nature of the reduction process. A cross-sectional view, obtained by folding the membrane (Figure 3b), clearly shows that the reduction is incomplete through the film’s vertical dimension. A thick, dense layer of unreacted GO remains at the bottom of the structure, confirming that the laser energy is attenuated as it penetrates the film. A side-view of the line’s edge (Figure 3c) further details the nonuniform vertical expansion. The unreacted GO base seen in Figure 3b represents a primary performance bottleneck for these structures as electrodes, as its lower conductivity will introduce high series resistance. While the surface is highly conductive, the buried GO layer introduces significant series resistance and acts as a reservoir for environmental moisture. To confirm the depth-control limits of our system, we increased the power to 3.6 W, which resulted in complete material excision and through-cutting of the membrane (Figure 3d,e). This indicates that while the laser *can* penetrate the full thickness, doing so leads to structural failure (cutting) rather than a fully reduced, integrated electrode. This structural compromise highlights the challenge of achieving through-thickness homogeneity without sacrificing the mechanical integrity of the free-standing film.

**Figure 3:**
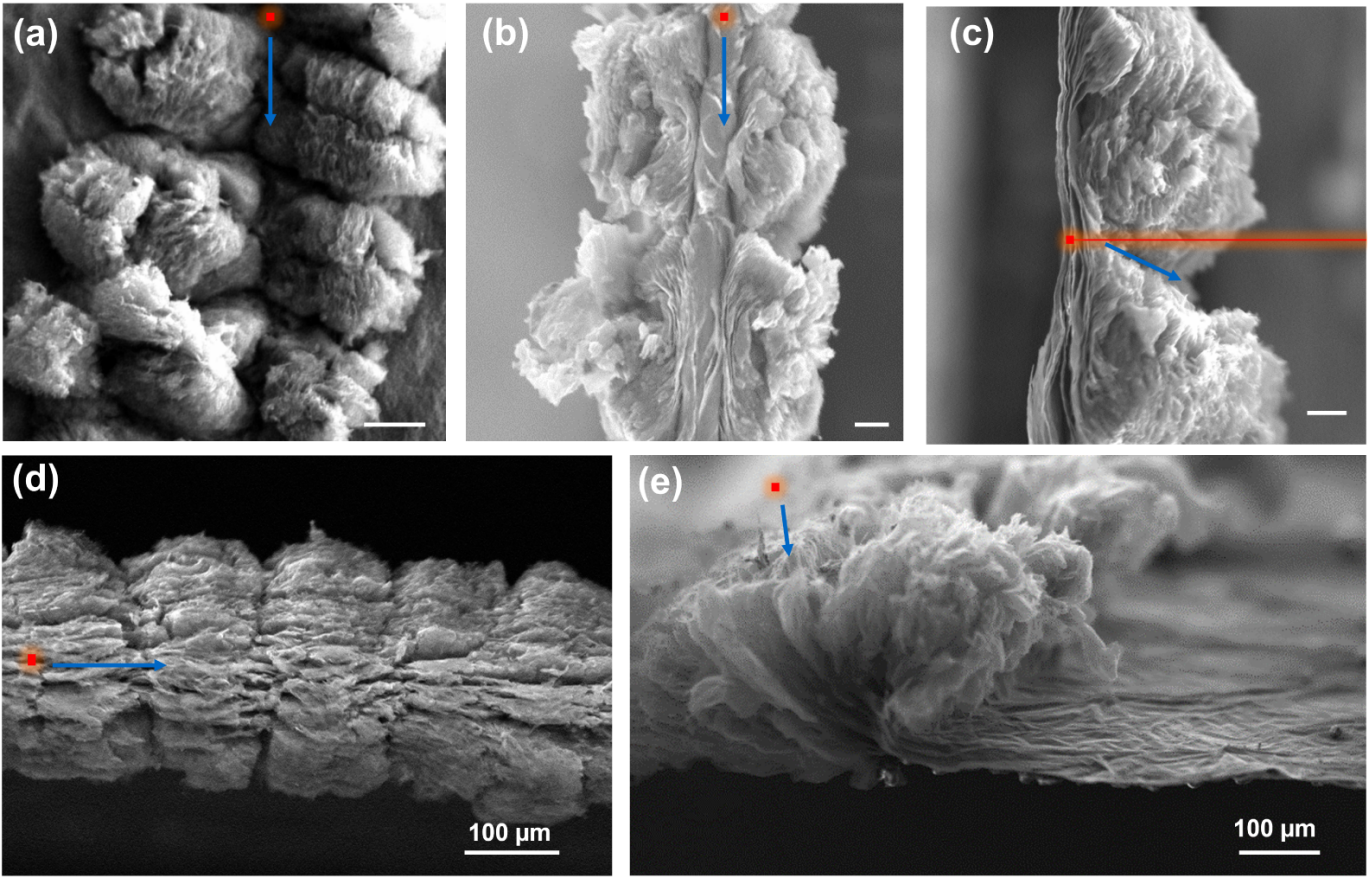
Morphological control and vertical heterogeneity of LrGO. (a) Top-view SEM image of continuous conductive lines formed by overlapping peony-like structures. (b) Cross-sectional SEM view of a folded membrane revealing the incomplete reduction and the dense, unreacted GO layer at the base. (c) Side-view SEM image detailing the vertical expansion profile at the line edge. (d) Cross-section and (e) side-view SEM images of a membrane fully penetrated (cut) using increased laser power (3.6 W), demonstrating the transition from surface patterning to material excision. Red dots and blue arrows indicate the laser focal point and scanning direction, respectively (scale bars: 100 μm).

### 3.2 Chemical and Structural Characterization of Laser-Reduced GO

To validate the changes in chemical composition and atomic structure, the material was analyzed before (pristine GO) and after laser treatment. Crucially, two different post-treatment samples were prepared to deconstruct the properties of the fully reduced material from the composite structure shown in Figure 4. The first sample, designated “LrGO”, consists of the as-is laser-patterned film, which includes both the porous LrGO top structure and the unreduced GO base. The second sample, designated “Pure LrGO”, was prepared by gently brushing off the top, porous LrGO structure, dispersing it in ethanol, and pressing it into a flat disk. This comparative analysis, presented in Figure 4, allows for a clear distinction between the properties of the fully reduced material and the bulk composite.

**Figure 4:**
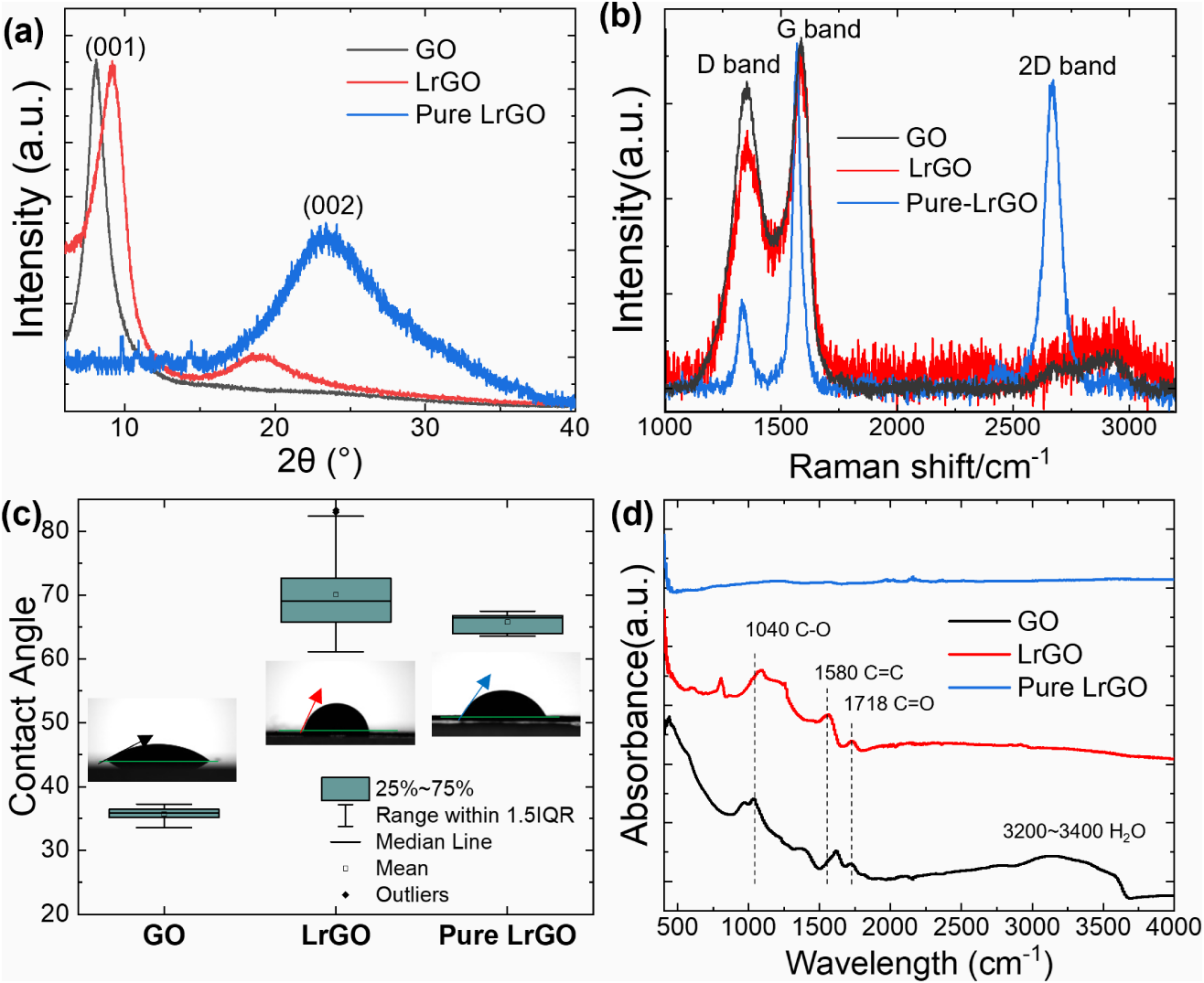
Structural, chemical, and surface energy characterization of GO and LrGO membranes. (a) XRD profiles of GO, LrGO, and pure LrGO. (b) Raman spectra showing D, G, and 2D bands; spectra are normalized to the G-band intensity. (c) Static water contact angle measurements (*n* = 5); box plots represent the median, mean (square), and 1.5 IQR. (d) FTIR spectra identifying characteristic oxygen-containing functional groups and C=C skeletal vibrations.

The XRD patterns (Figure 4a) effectively illustrate the changes in the interlayer distance (*d*-spacing) of the materials. Pristine GO exhibits a sharp diffraction peak at 2θ ≈ 8.1°, corresponding to a large *d*-spacing of approximately 1.09 nm. This large separation is characteristic of GO, where bulky OFGs and intercalated water molecules separate the carbon sheets.^28,29^ The LrGO sample shows a right shift in this peak to 2θ ≈ 9.2°, corresponding to a decreased *d*-spacing of approximately 0.96 nm. The presence of this rightward shift peak, along with small bump around 19°, provides direct evidence of incomplete reduction, as some dehydration and partial removal of OFGs occurred, but the graphene sheets did not fully restack. In contrast, the pure LrGO sample shows a complete disappearance of the GO peak. It exhibits a broad peak centered at 2θ ≈ 23.2°, which corresponds to a *d*-spacing of approximately 0.38 nm. This value is much closer to the *d*-spacing of graphite (∼0.34 nm), and the peak’s broadening is characteristic of turbostratic carbon structures.^30^ This significant decrease in *d*-spacing is direct evidence of the effective removal of OFGs and water, allowing the graphene sheets to restack more closely.

Raman spectroscopy (Figure 4b) was used to probe the carbon lattice structure. All samples show the characteristic D-band (∼1350 cm^–1^) and G-band (∼1590 cm^–1^), which correspond to defects and the in-plane vibration of sp^2^-hybridized carbon atoms, respectively. Pristine GO exhibits an intensity ratio of the D to G band (*I*_*D*_/*I*_*G*_) of 0.86, reflecting the high density of OFGs disrupting the hexagonal lattice. The as-treated LrGO shows a slight decrease to 0.76; however, this value is a composite measurement influenced by the underlying, unreduced GO bulk due to the laser’s penetration profile. In contrast, the pure LrGO (recollected surface layer) displays a significantly lower *I*_*D*_/*I*_*G*_ of 0.25. This is consistent with the findings of Grote et al., who demonstrated that controlled thermal processing can promote the reorganization of OFGs to leave behind an intact hexagonal lattice with minimal defects.^31^ This marked decrease, coupled with the emergence of a prominent 2D-band (∼2670 cm^–1^), indicates that the laser treatment successfully restores large sp^2^ domains and promotes graphitic restacking. While many reduction methods increase the *I*_*D*_/*I*_*G*_ ratio by creating numerous small, isolated sp^2^ flakes, our laser process facilitates the “healing” of the lattice, resulting in a more ordered, crystalline structure.^31^

The removal of hydrophilic OFGs should render the material more hydrophobic, a transition confirmed by contact angle measurements (Figure 4c). Pristine GO is hydrophilic, with an average water contact angle of 36°. In contrast, the laser-treated LrGO sample is hydrophobic, with a much higher average contact angle of 69°. Interestingly, the flat disk of pure LrGO has a slightly lower contact angle (67°) but with significantly less variation. The higher angle and large standard deviation (±11°) of the as-is “LrGO” sample can be attributed to its porous, peony-like surface topography, which introduces a roughness effect that amplifies its inherent hydrophobicity.^32^

FTIR spectroscopy (Figure 4d) provides the most direct confirmation of the top-down reduction effect. The pristine GO spectrum (black trace) shows numerous strong absorption peaks corresponding to a wide variety of OFGs, such as C=O (1718 cm^–1^), CO-H bend (1400 cm^–1^, br), C–OH stretch (1040 cm^–1^), epoxy stretch (970 cm^–1^) and a broad O–H stretch (∼3600 cm^–1^, sh). In stark contrast, the pure LrGO spectrum (blue trace) is nearly featureless, confirming the highly efficient removal of almost all OFGs from the top layers of the material. Crucially, the as-is LrGO sample (red trace) reveals the evolution of the original GO toward the fully reduced material. Loss of OFGs is revealed by the decreased intensity of peaks at 1718, 1400, and 970 cm^–1^. The reduction of OFGs also leads to a loss of water from the structure as evidenced by the substantial decrease in intensity of both the water stretching (3200–3400 cm^–1^, br) and bending (1620 cm^–1^) vibrations. Simultaneously, the shoulder near 1580 cm^–1^ in the GO spectrum corresponding to stretching of C=C functional groups grows in as a prominent peak in the LrGO spectrum. Its absence in the pure LrGO spectrum is attributed to the highly symmetric structure of the fully reduced material, which decreases the intensity of this IR transition. The sharp peak that appears at 810 cm^–1^ in the LrGO spectrum may be tentatively assigned to C=C–H bending vibrations, which have intensities that are similarly sensitive to the local symmetry of the chemical environment. The FTIR data reveals additional complexity in the chemical evolution from reduction as a broad, nearly featureless band grows in from 1050 to 1250 cm^–1^, which would be challenging to assign to any specific functional groups.^33^

The survey scan of the pristine GO film (Figure 5a) shows the presence of primarily carbon and oxygen, with an initial atomic oxygen-to-carbon (O/C) ratio of approximately 0.45. The high-resolution C 1s spectrum of GO (Figure 5c) can be deconvoluted into three main components: the main graphitic C–C/C=C peak (284.7 eV), a significant contribution from hydroxyl/epoxy groups (C–O at 286.8 eV), and a smaller peak from carbonyl groups (C=O at 288.1 eV).^29^ The corresponding O 1s spectrum (Figure 5d) reinforces this, showing peaks for C–O (∼533 eV) and C=O (∼531.5 eV) bonds. The large intensity of the C–O peak highlights the highly oxidized state of the initial material, with a C 1s (C–O) component of 24.55% as shown in Table 1.

**Figure 5:**
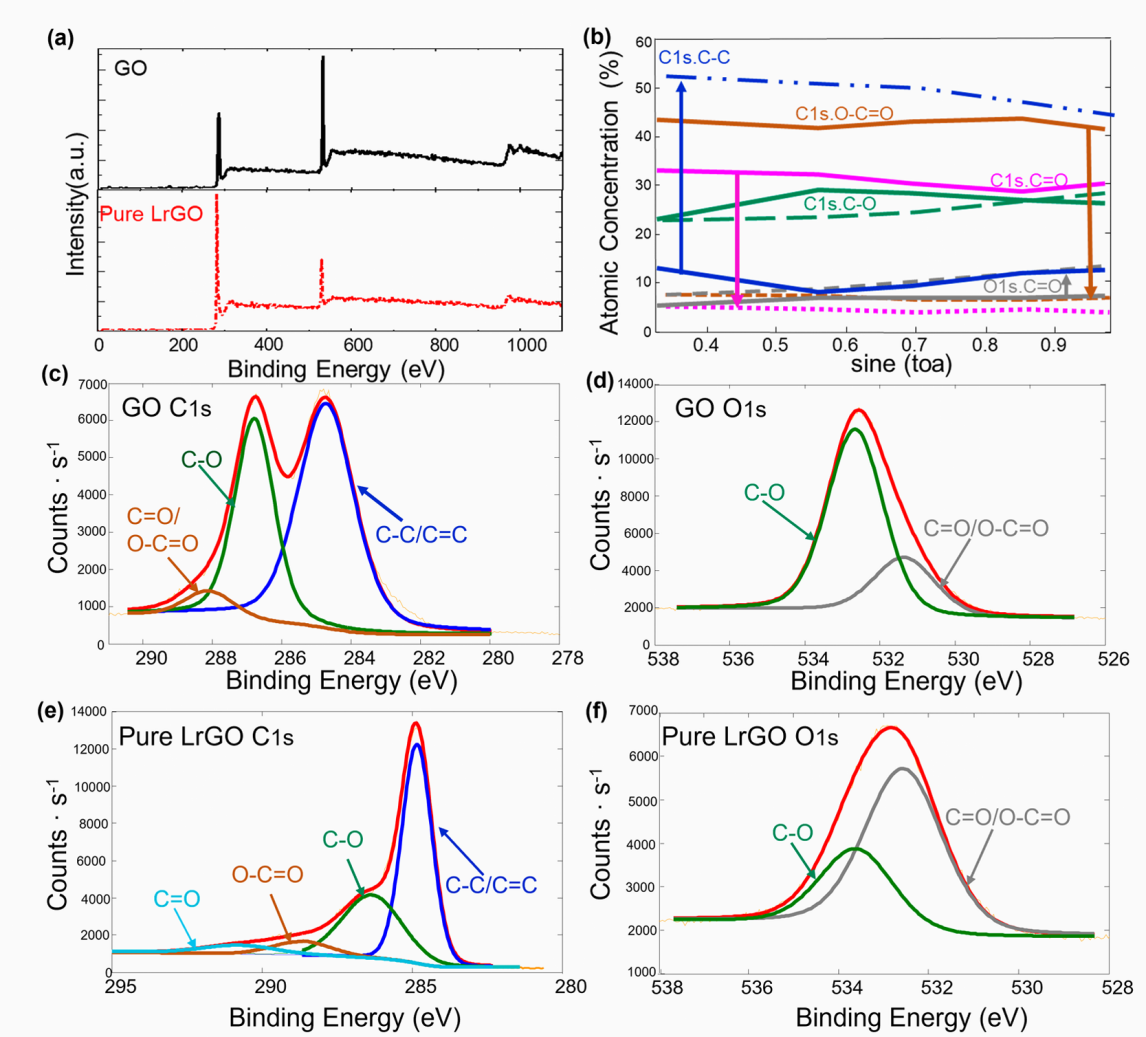
XPS analysis of the surface chemistry of GO and pure LrGO. (a) Survey scan XPS spectrum of the pristine GO film. (b) Angle-resolved atomic concentration of different carbon and oxygen species for pristine GO (solid lines) and pure LrGO (dashed lines), showing the chemical composition as a function of depth. High-resolution, deconvoluted spectra of the (c) C 1s and (d) O 1s regions for GO, and the (e) C 1s and (f) O 1s regions for pure LrGO.

**Table 1:** XPS Atomic Percentage of Various Carbon and Oxygen Species for GO and pure LrGO

Sample	C^1s^(C–C)	C^1s^(O–C)	C^1s^(O–C=O)	C^1s^(C=O)	O^1s^(C–O)
GO	41.79	24.55	2.98	-	10.24
Pure LrGO	54.34	17.87	7.23	5.42	7.94

After the laser treatment, the chemical nature of the surface is dramatically altered. The high-resolution C 1s spectrum of pure LrGO (Figure 5e) is now dominated by a sharp, intense sp^2^ -hybridized C=C peak at 284.8 eV. Concurrently, the peaks associated with oxygen functional groups (C–O and C=O) are substantially diminished, as seen in the quantitative analysis (Table 1). The C 1s (C–C) component increases from 41.79% to 54.34%, while the C 1s­(C–O) and O 1s­(C–O) components decrease from 24.55% and 10.24% to 17.87% and 7.94% respectively. This indicates the successful removal of these groups and the restoration of the graphitic carbon lattice. This is corroborated by the pure LrGO O 1s spectrum (Figure 5f), which shows a significant overall decrease in intensity and a shift in peak positions, confirming the removal of oxygen.

The most compelling evidence for the reduction mechanism comes from the angle-resolved XPS (ARXPS) data shown in Figure 5b. This technique provides a nondestructive depth profile of the material’s chemistry, where smaller takeoff angle (smaller sine (θ)) is more sensitive to the top surface, and larger angle (sine­(θ) approaching 1) probe deeper into the film. For the pristine GO film (solid lines), the atomic concentrations of the different chemical species are relatively constant across all angles, indicating a chemically uniform, bulk material. In stark contrast, the pure LrGO (dashed lines) exhibits a strong chemical gradient with depth. At the outermost surface (low sine­(θ)), the concentration of graphitic carbon (C=C, C–C) is at its maximum (∼55%), while the oxygenated species (C–O, C=O) are at their minimum. As the analysis probes deeper into the film (increasing sine­(θ)), the concentration of graphitic carbon steadily decreases, while the concentration of the oxygenated species increases. This result is the definitive chemical confirmation of the “top–down” reduction process observed in the cross-sectional SEM images (Figure 3). The laser energy is most effective at the surface, leading to nearly complete reduction, and its effect diminishes with depth, leaving behind a more oxidized, GO-like material at the base.

The porous structure of LrGO is crucial for the sensing performance of LrGO-based sensors, with nanometer-level porosity often playing a more significant role than micrometer-level pores. To understand the internal porous architecture of the pure LrGO material, nitrogen adsorption–desorption measurements were conducted. This technique effectively probes pores ranging from approximately 1.7 to 300 nm in diameter. It is important to note that larger, micrometer-sized macropores—like those observed via SEM—are beyond the detection range of this method, so this analysis primarily reflects the mesoporous features. Attempts to obtain similar data for GO membranes were unsuccessful, likely due to their tightly packed, lamellar structure, which restricts nitrogen gas permeation.

The adsorption–desorption isotherm for pure LrGO (Figure 6a) exhibits a Type IV curve with a H3 hysteresis loop.^34^ This is characteristic of aggregates of plate-like particles that give rise to slit-shaped pores or networks of open, large pores, a morphology consistent with the laminar or lamellar internal structure of graphene-based materials. The high volume of adsorbed nitrogen clearly indicates a well-developed mesoporous network.

**Figure 6:**
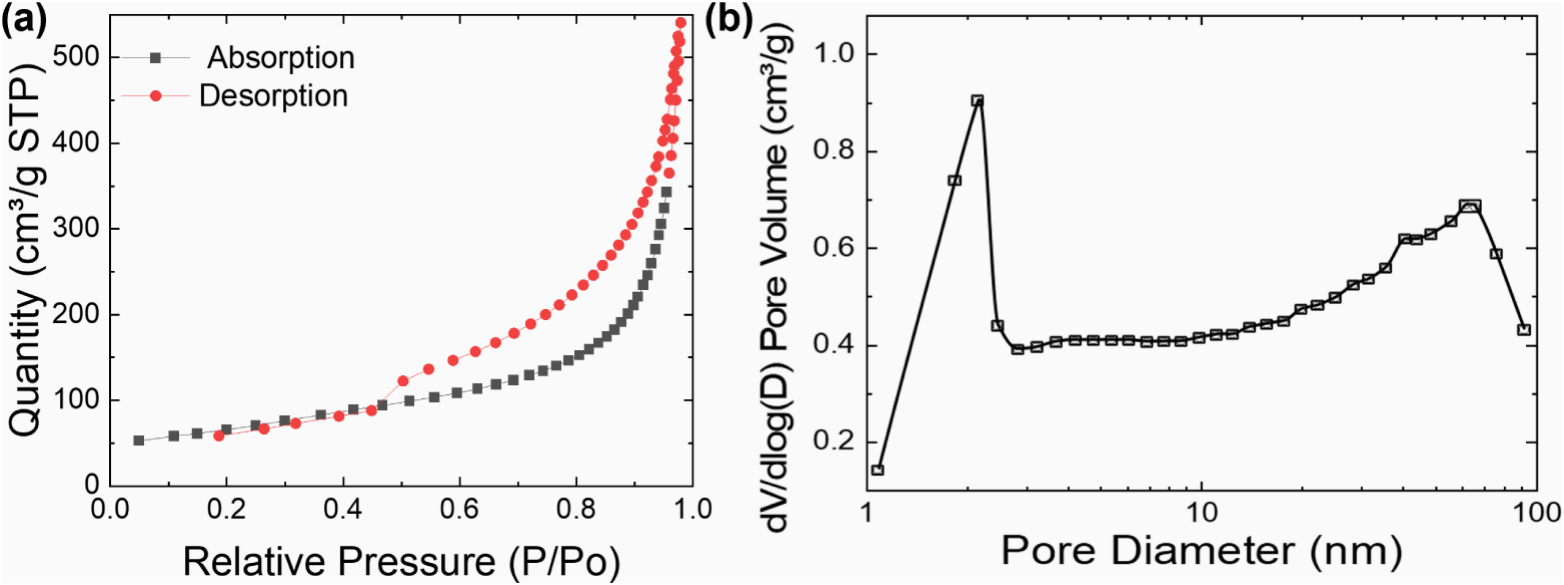
Nitrogen adsorption/desorption isotherms (a) and their pore size distribution (b) of typical pure LrGO.

The profound impact of the laser-induced photothermal process is further underscored by quantitative observations. Pure LrGO has a substantial BET surface area (SBET) of 234.09 m^2^/g, with total cumulative pore volumes of 0.82 cm^3^/g (adsorption) and 0.84 cm^3^/g (desorption). The BJH desorption average pore diameter is calculated to be 10.83 nm, reflecting the broad range of mesopores generated during the reduction.

The pore size distribution plot (Figure 6b) reveals a bimodal distribution with a predominant peak in the 2–15 nm range and another significant contribution from pores around 10.83 nm. The larger cumulative pore volume observed in the desorption branch further supports the increased prevalence of these nanosized pores. Therefore, the larger pores observed in the SEM images (Figures 1–3) are evidently filled with a high density of these smaller, interconnected mesopores, demonstrating that the laser-induced reduction process is highly effective in generating a significantly enhanced nanosized porous network.

### 3.3 Humidity-Dependent Impedance of LrGO Electrodes

Given the highly porous structure and large surface area, the potential of the laser-patterned LrGO for humidity sensing was investigated. The electrochemical impedance was measured as a function of relative humidity (RH), with the results presented in Figure 7. The Bode plot (Figure 7a) shows that at any given frequency, the impedance systematically decreases as ambient humidity increases. This is attributed to the adsorption of water molecules onto the LrGO surface, which provides pathways for protonic conduction (H^+^ hopping), creating a parallel conduction path that lowers the overall impedance.

**Figure 7:**
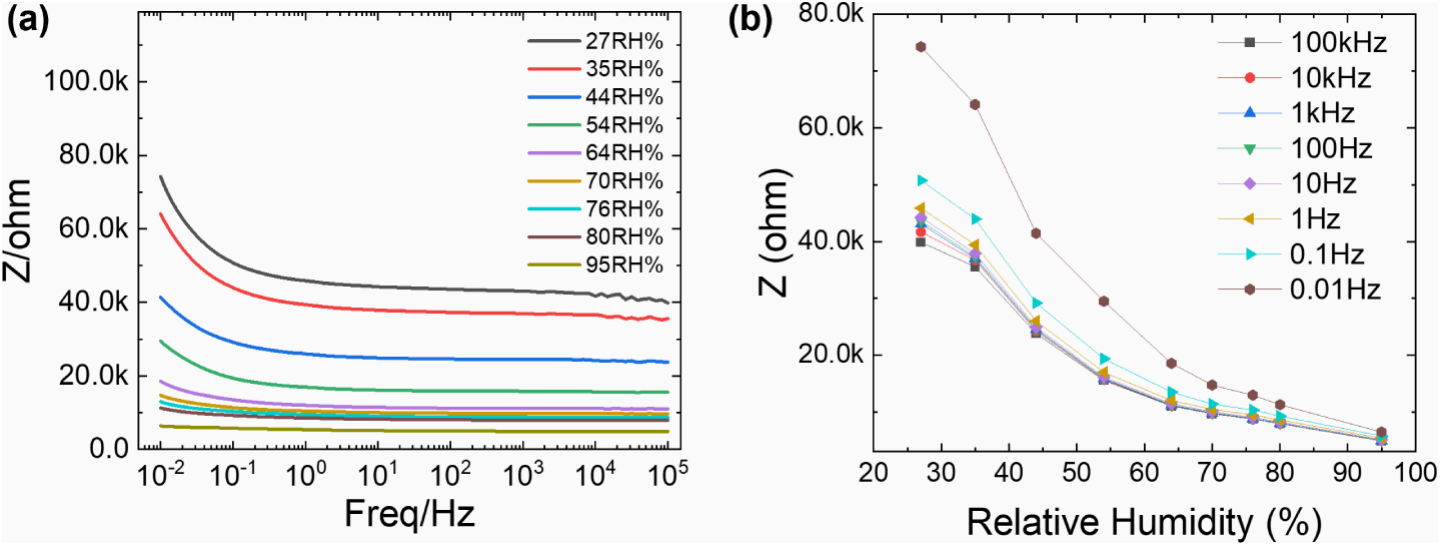
Humidity-dependent impedance characteristics of the LrGO electrode. (a) Bode plot of impedance magnitude versus frequency at various relative humidity (RH) levels from 27% to 95%. (b) Impedance magnitude as a function of RH plotted for discrete frequencies, illustrating the material’s sensitivity as a humidity sensor.

As shown in Figure 7b, the device exhibits a strong, clear response, with impedance dropping by over an order of magnitude across the tested RH range, particularly at low frequencies where protonic conduction dominates. The Nyquist plot (Figure S2) further supports this, showing that the charge transfer resistance (Rct) decreases dramatically with increasing RH. These initial results suggest that the porous, laser-induced LrGO architecture is a promising candidate for fabricating sensitive humidity sensors.

To evaluate the practical sensor viability of the heterogeneous LrGO structure, its dynamic response (normalized impedance change) to humidity was tested. As shown in Figure 8, the electrode exhibits a clear decrease in impedance as RH increases, with a significant negative baseline drift and peak response degradation over repeated cycles. The sensor fails to reach a stable plateau during the exposure phase, indicating a diffusion-limited response rather than a rapid surface-adsorption mechanism.

**Figure 8:**
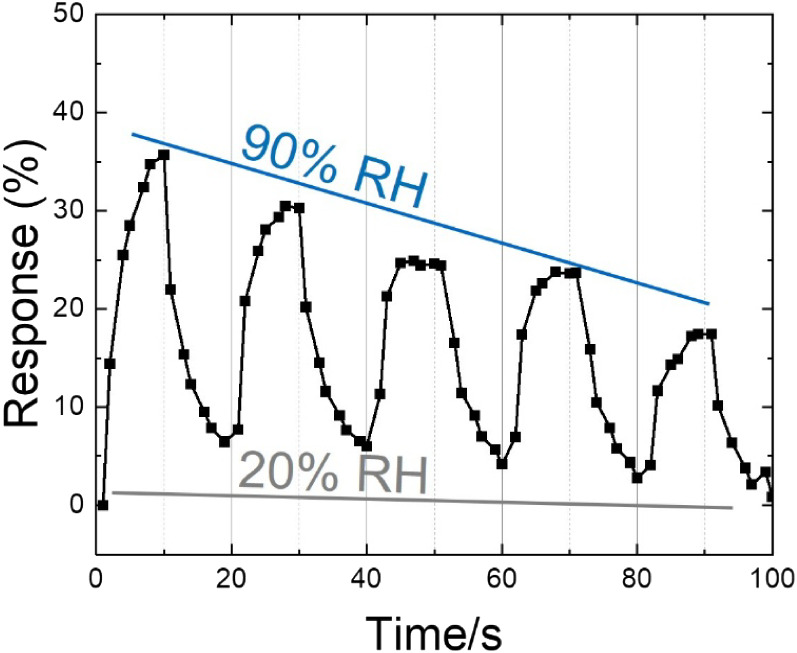
Humidity sensing. Typical response and recovery curves to a RH% between 20% and 90%.

Most notably, the recovery phase is characterized by a sluggish and incomplete return to the baseline; full recovery requires several days in a desiccated environment. We attribute this to incomplete desorption and moisture accumulation within the gradient of oxygen functional groups identified in our depth profiles. Residual OFGs in the less-reduced regions may act as high-affinity “traps” where water molecules are absorbed into the bulk rather than merely adsorbing on the surface.^35^ This internal trapping mechanism is compounded by charge trapping at the LrGO/GO interface, where the underlying hydrophilic reservoir creates a metastable electrical environment. Consequently, while the porous LrGO morphology is visually promising, its inherent through-thickness heterogeneity makes it unsuitable as a standalone sensing electrode. This underscores the fundamental limitation of direct laser writing: the top-down reduction leaves a “buried” hydrophilic reservoir that compromises device stability.

To further probe the general viability of this vertically heterogeneous structure for sensing applications, its mechanical stability and response were also evaluated. The unique porous structure of LrGO, with its interconnected micrometer- and nanometer-sized pores, suggests potential for mechanical sensing applications.^36–38^ However, as demonstrated by the inconsistent humidity sensing, the material’s structural integrity is a potential weakness. The sensor’s response to a cyclic pressure load (50 g) was monitored by calculating the normalized impedance change, or response, over time (Figure 9b). The data reveals a characteristic initial response: upon application of pressure, the response increases sharply, which is consistent with the elastic compression of the porous dielectric network. However, the lack of mechanical-dielectric robustness is immediately apparent in the subsequent cycles. The baseline fails to return to zero, and the peak response magnitude diminishes rapidly. This “signal fatigue” confirms that the vertically heterogeneous structure cannot maintain a stable capacitive interface, as the structural “gluing” provided by the original OFGs has been removed, leaving the delaminated “peony” layers prone to irreversible mechanical shifting.

**Figure 9:**
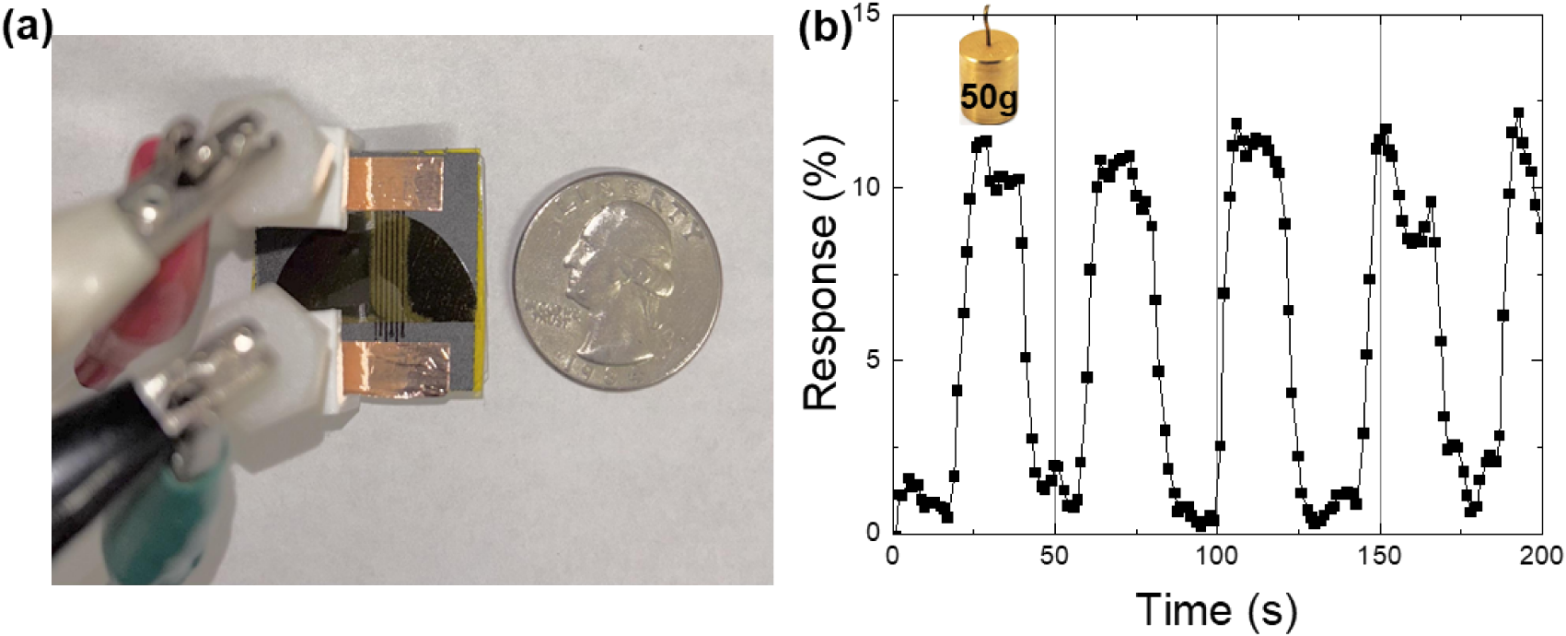
Pressure sensing characterization of LrGO. (a) Photograph of the LrGO pressure sensor setup. (b) Dynamic impedance response of the LrGO sensor to cyclic pressure loading.

### 3.4 Structural Evolution and Mechanistic Limitations

The pulsed CO_2_ laser provides localized photothermal energy that facilitates the structural restoration of the GO lattice. Unlike bulk thermal reduction, which often results in an amorphous or ruptured carbon structure, this laser-driven pathway favors thermal disproportionation. As noted by Grote et al., this mechanism promotes the formation of ordered sp^2^ graphene domains adjacent to point defects, effectively “healing” the hexagonal framework.^31^ This explains the high crystallinity (low I_D_/I_G_ and prominent 2D band) observed in our pure LrGO samples.

The functional performance of the irradiated membranes is governed by the through-thickness heterogeneity inherent to the top-down reduction process. Due to the high absorption coefficient of GO at 10.6 μm, the laser intensity follows an exponential decay according to the Beer–Lambert Law. Consequently, the deoxygenation threshold is surpassed only in the top 1–3 μm, while the bulk interior remains largely pristine. This effect is amplified by the increased opacity of the newly formed rGO “skin,” which provides an optical shielding effect that protects the underlying dielectric core. While this gradient architecture is essential for maintaining mechanical flexibility, it represents a local optimum rather than a global one. Our parametric study (one-factor-at-a-time, OFAT, approach) highlights that even at 2.4 W, this heterogeneity is an intrinsic limitation of the material’s optical attenuation rather than a lack of parameter tuning. However, improved overall material performance may be attainable through a systematic study of all laser parameters (scan speed, modulation frequency, spot size, etc.) involved in material processing.

The presence of this “buried” hydrophilic reservoir is the primary driver for the observed sensor instability. The negative baseline drift and gradual reduction in peak response over repeated cycles are characteristic of incomplete desorption and moisture accumulation. In our device, water molecules diffuse into the deep-seated pores of the peony-like architecture, where they become trapped by the high-affinity residual OFGs in the unreduced bulk. This accumulation, coupled with charge trapping at the LrGO/GO interface, creates a screening effect that dampens the electrical responsiveness and prevents the impedance from returning to its initial state. This underscores that while the laser-induced morphology is promising, the vertical chemical gradient acts as a functional bottleneck for reliable, long-term sensing applications.

## 4 Conclusion

In summary, we have demonstrated that pulsed CO_2_ laser reduction is a powerful tool for the rapid, single-step fabrication of porous carbon electrodes, with a locally optimized power of 2.4 W yielding a high-quality peony-like morphology (234 m^2^/g). Structural and chemical analyses confirm that this process follows a thermal disproportionation pathway, effectively healing the graphene lattice and achieving a significantly lower *I*_*D*_/*I*_*G*_ ratio of 0.25.

However, our investigation reveals a fundamental limitation intrinsic to the top-down laser writing process. Due to the high optical attenuation of GO at 10.6 μm, the reduction is confined to a thin surface “skin” (1–3 μm), leaving behind a “buried” hydrophilic reservoir of unreduced bulk material. This through-thickness heterogeneity is the primary driver of device instability, where moisture accumulation and charge trapping result in significant baseline drift and sensitivity degradation over time.

These findings underscore that while laser-induced reduction can restore high-quality surface crystallinity, achieving commercial-grade stability will require strategies to mitigate this internal gradient, such as dual-side irradiation or the optimization of membrane thickness. This study provides a mechanistic framework for future developments in reliable, laser-scribed 2D electronics.

## Supplementary Material



## Data Availability

The authors declare that the data supporting the findings of this study are available within the paper and supplement.

## Notes

Typical SEM image and elemental analysis of LrGO; electrochemical impedance spectroscopy of LrGO; cyclic voltammetry curves of LrGO microsupercapacitor under varying relative humidity (PDF)
