# Supplementary material for: Fundamental Limitation: How Through-Thickness Heterogeneity in Laser-Reduced Graphene Oxide Compromises Sensor Stability
Source: ACS Appl Nano Mater. 2026 May 7;9(20):9409–19. doi: 10.1021/acsanm.6c00918 (PMC13200162; doi:10.1021/acsanm.6c00918)
Supplement: Supplementary file 1 [file an6c00918_si_001.pdf]

# Supporting Information

# Fundamental Limitation: How Through-Thickness Heterogeneity in Laser-Reduced Graphene Oxide Compromises Sensor Stability

*Ryan Russell[1], Alex Medeiros[1], Aiden Rowley[1], Sandra Schujman[2], Ivan V. Vlassiouk[3], Timothy J. Barnum[4], Yijing Stehle[1*]*

1 Department of Mechanical Engineering, Union College, Schenectady, NY 12308, USA

2 NYCREATES, Albany, NY 12203, USA

3 Center for Nanophase Materials Sciences, Oak Ridge National Laboratory, Oak Ridge, TN, 37831, USA

4 Department of Chemistry, Union College, Schenectady, NY 12308, USA

*Corresponding author: stehley@union.edu

**Table of contents**

**Figure S1.** Typical SEM image and their Carbon (red, middle), oxygen (green, right) of laser-induced rGO with laser power of (a )2.4w, (b)3w, (c)3.6w. **3**

**Figure S2.** Electrochemical Impedance Spectroscopy (EIS) of LrGO. **4**

**Figure S3.** Environmental sensitivity of LrGO micro-supercapacitors. Cyclic voltammetry (CV) curves of a laser-scribed concentric MSC recorded at a scan rate of 100mV/s under varying relative humidity (RH). **5**

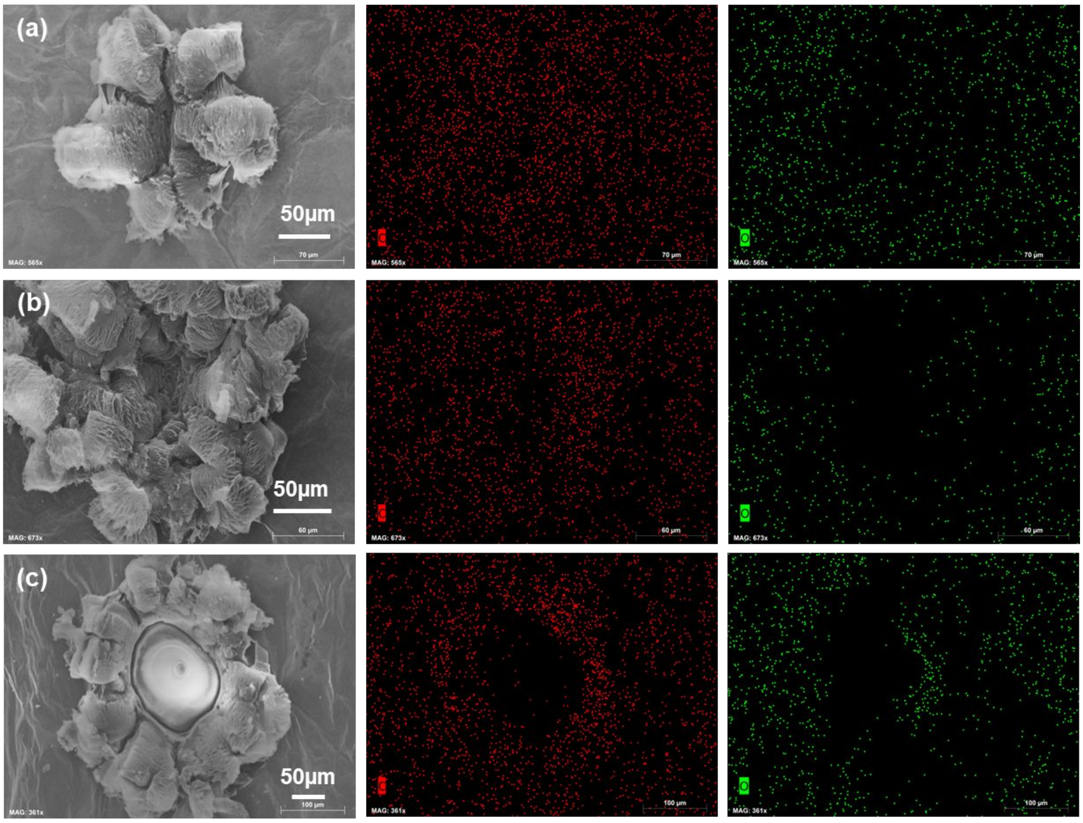


**Figure S1.** Typical SEM image and their Carbon (red, middle), oxygen (green, right) of laser-induced rGO with laser power of (a )2.4w, (b)3w, (c)3.6w.

Fig. S1a-c shows SEM image and the corresponding EDX maps for carbon(green) and oxygen(blue) for the laser condition has the highest potential of get lowest O/C ratio. According to Fig. S1a with laser power of 2.4w, there is not an obvious carbon vanishing around the laser dot, but an O atom decreasing around the laser dot can be identified. The drastically decrease of O/C ratio from the GO region (~0.4) to the rGO region (~0.2) indicates successful removal of the OFGs through laser reduction at the power of 2.4w (Fig. S1a). It is also obvious that the reduction is not 100%, there is still plenty of unreduced OFGs and even GO membrane on the bottom, which could greatly influence the effective conductivity of reduced rGO. However, further increase the laser power does not further decrease the O/C ratio. As shown in Fig. S1b, laser beam with power of 3w show an empty O atom region around the laser dot, but there start to have C atom vanishing, which does not lead to a O/C ratio decrease. Further increase laser power to 3.6w as shown in Fig. S1c, both C and O vanish around laser dot can be identified and O/C ratio

increase up to 0.5. At 3.0 W (Fig. S1b), the onset of preferential carbon ablation is observed. The slight increase in the relative oxygen atomic percentage at this power is attributed to the photothermal volatilization of the $sp^2$ carbon framework rather than chemical re-oxidation. At 3.6 W (Fig. S1c), the energy density surpasses the ablation threshold of the entire membrane, leading to complete material removal (through-holes).

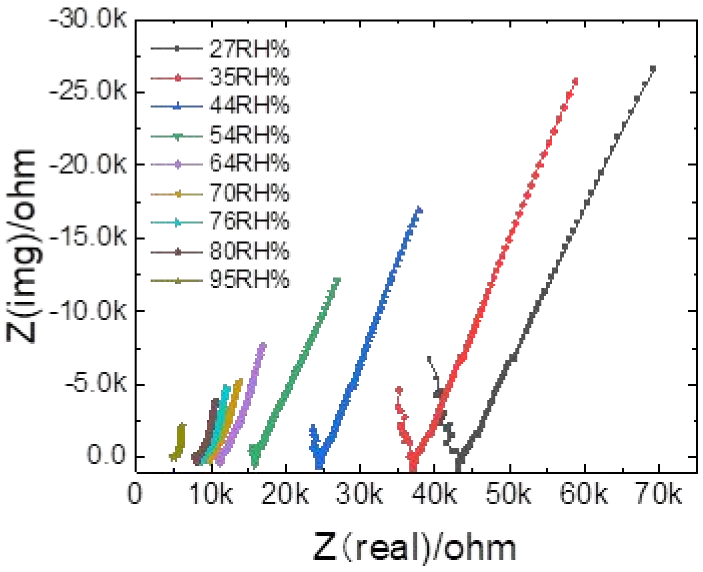


**Figure S2.** Electrochemical Impedance Spectroscopy of LrGO.

The Nyquist plot demonstrates the frequency-dependent complex impedance of the laser-reduced membrane. The plot features a characteristic semi-circle in the high-frequency region, corresponding to the charge-transfer resistance at the electrode interface, followed by a linear Warburg impedance tail in the low-frequency region. The relatively low charge-transfer resistance indicates that while the LrGO possesses through-thickness heterogeneity and associated charge trapping compromise sensor stability. the surface reduction is sufficient to facilitate efficient charge transport, supporting its use in the sensing and supercapacitor applications discussed in the text.

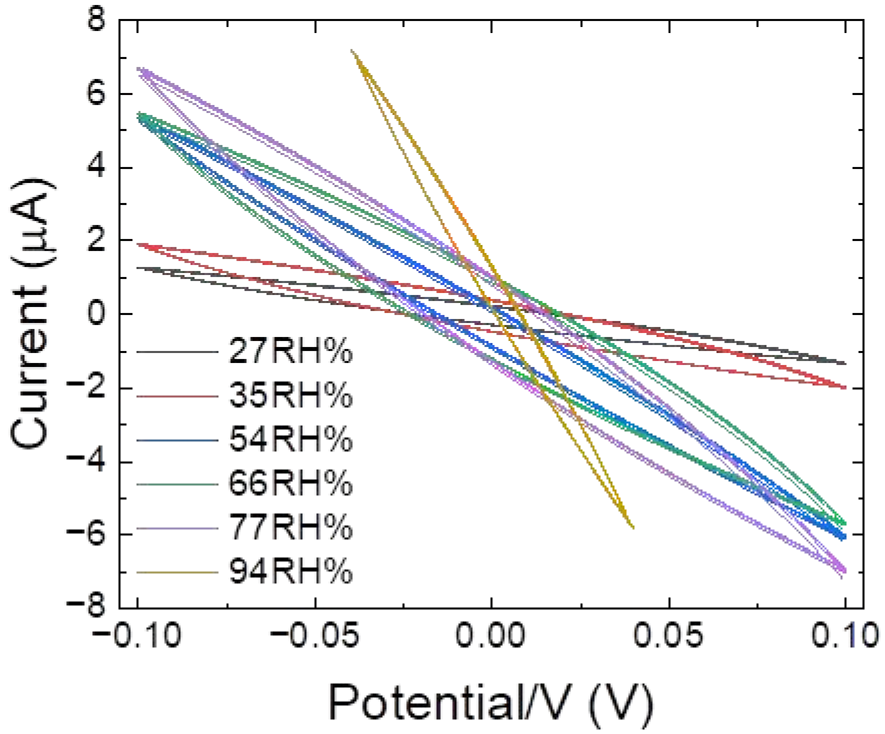


**Figure S3.** Environmental sensitivity of LrGO micro-supercapacitors. Cyclic voltammetry (CV) curves of a laser-scribed concentric MSC recorded at a scan rate of 100mV/s under varying relative humidity.

The electrochemical performance of the LrGO micro-supercapacitor (MSC) displays a strong dependence on environmental moisture. At lower humidity, the CV curves exhibit a narrow, tilted profile characteristic of a highly resistive capacitive system. As humidity increases, the slope of the CV curves increases significantly, indicating a reduction in the equivalent series resistance (ESR). This trend suggests that adsorbed water molecules within the "peony-like" porous architecture facilitate ion transport, effectively enhancing the charge-transfer kinetics.

Notably, the curves maintain a featureless, quasi-rectangular shape without significant redox peaks, confirming that the energy storage mechanism remains dominated by electric double-layer capacitance (EDLC) rather than faradaic (pseudo-capacitive) reactions. While the increased humidity improves the apparent electrochemical response, it highlights the device's lack of environmental stability, as the baseline performance is dictated by ambient conditions rather than intrinsic material properties. The lack of environmental stability in the LrGO MSC is a direct

consequence of moisture accumulation within the 'peony-like' architecture. As water molecules occupy the high-affinity sites of the unreduced GO core, they establish a metastable conductive network. This leads to the negative baseline drift observed in sensing cycles, as the incomplete desorption of these trapped molecules prevents a return to the initial state.
